# Egr-1 is involved in coronary microembolization-induced myocardial injury via Bim/Beclin-1 pathway-mediated autophagy inhibition and apoptosis activation

**DOI:** 10.18632/aging.101616

**Published:** 2018-11-04

**Authors:** Xian-tao Wang, Xiao-dan Wu, Yuan-xi Lu, Yu-han Sun, Han-hua Zhu, Jia-bao Liang, Wen-kai He, Lang Li

**Affiliations:** 1 Department of Cardiology, the First Affiliated Hospital of Guangxi Medical University, Nanning 530021, Guangxi Zhuang Autonomous Region, China; * Equal contribution

**Keywords:** coronary microembolization, Egr-1, autophagy, apoptosis, myocardial injury

## Abstract

Coronary microembolization (CME) substantially reduces the clinical benefits of myocardial reperfusion therapy. Autophagy and apoptosis participate in the pathophysiological processes of almost all cardiovascular diseases, including CME-induced myocardial injury, but the precise underlying mechanisms remain unclear. In the present study, we observed that Egr-1 expression was substantially increased after CME modeling. Inhibition of Egr-1 expression through the targeted delivery of rAAV9-Egr-1-shRNA improved cardiac function and reduced myocardial injury. The microinfarct size was also significantly smaller in the Egr-1 inhibitor group than in the CME group. These benefits were partially reversed by the autophagy inhibitor 3-MA. As shown in our previous study, autophagy in the myocardium was impaired after CME. Inhibition of Egr-1 expression in vivo restored the autophagy flux and reduced myocardial apoptosis, at least partially, by inhibiting the Egr-1/Bim/Beclin-1 pathway, as evidenced by the results of the western blot, RT-qPCR, and TUNEL staining. At the same time, TEM showed a dramatic increase in the number of typical autophagic vacuoles in the Egr-1 inhibitor group compared to the CME group. Based on these findings, the Egr-1/Bim/Beclin-1 pathway may be involved in CME-induced myocardial injury by regulating myocardial autophagy and apoptosis, and this pathway represents a potential therapeutic target in CME.

## Introduction

Coronary microembolization (CME) is a common complication in patients with acute coronary syndrome (ACS) during percutaneous coronary intervention (PCI), which could be caused by microvascular obstructions with atherosclerotic plaques, microthrombus or neutrophils-platelet aggregates [1–3]. CME is one of the most important causes of the no-reflow phenomenon, which is responsible for the loss of clinical benefits from myocardial reperfusion therapy [4,5]. CME affects the long-term clinical follow-up of individuals with ACS mainly because of myocardial contractile dysfunction and notable arrhythmias [6–8]. The prediction and prevention of CME have been difficult challenges for cardiovascular intervention physicians. As shown in our previous study, myocardial autophagy may play regulative role in CME-induced myocardial injury [9]. However, the specific molecular mechanism remains unclear.

Autophagy is widely involved in the pathogenesis and development of cardiovascular diseases [10–13]. In our previous study, we identified several mRNAs that were remarkably dysregulated in the rat myocardium after CME using a microarray analysis. Among those markedly upregulated mRNAs, Egr-1 has a key function in myocardial cell injury [14,15]. Some research is making progress in identifying the mechanism by which the Egr-1/Bim pathway regulates autophagy and apoptosis [16–18]. However, the roles of the Egr-1/Bim pathway in myocardial injury and cardiac dysfunction after CME remain unknown.

In the present study, we hypothesized that the Egr-1/Bim/Beclin-1 pathway is associated with CME-induced myocardial injury by controlling myocardial autophagy and apoptosis. We established a rat CME model to investigate the activation of the Egr-1/Bim/Beclin-1 pathway after CME and its specific mechanism in mediating myocardial injury.

## RESULTS

### Rat CME model

**Figure 1:**
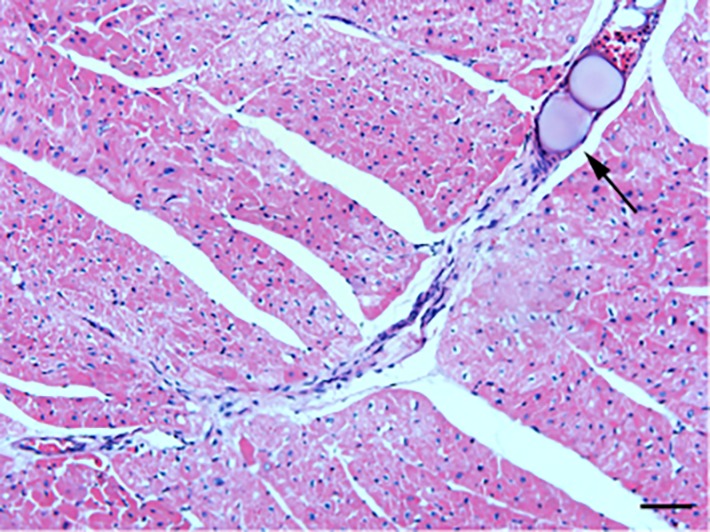
**HE staining of microinfarct areas after CME modeling.** Myocardial tissue exhibited edema and degeneration around the microspheres, accompanied by a large number of infiltrating inflammatory cells. The arrow indicates microspheres in an arteriole (x200, scale bar = 50 μm).

HE staining was conducted to detect the histological changes in the myocardial tissue of rats with CME. As shown in Fig.1, myocardial edema was observed around the microspheres, accompanied by a large amount of accumulated inflammatory cells.

### Inhibition of Egr-1 improved cardiac function following CME

Cardiac dysfunction was induced by CME compared to the Sham group. As shown in Table 1, LVEF, LVFS, and CO were decreased, while LVIDd was increased (*P* < 0.05). Egr-1 inhibition significantly improved cardiac function following CME compared to the CME group (*P* < 0.05). The benefit was decreased by the autophagy inhibitor 3-MA. Based on these results, Egr-1 inhibition improves cardiac function at least partially by upregulating autophagy in this rat model of CME.

**Table 1:** Cardiac function parameters of rats in each group following CME modeling.

Group	*N*	LVEF(%)	LVFS(%)	CO(L/min)	LVIDd(mm)
Sham	6	83.96±3.31	53.38±2.47	0.25±0.018	5.83±0.46
CME	6	53.76±4.20^a^	25.43±1.51^a^	0.10±0.016^a^	7.46±0.45^a^
CME+shRNA	6	74.33±5.49^ab^	47.84±3.42^ab^	0.21±0.027^ab^	6.29±0.38^ab^
CME+Control	6	52.15±4.17^ac^	25.77±2.03^ac^	0.11±0.016^ac^	7.44±0.54^ac^
CME+shRNA+3-MA	6	64.11±4.55^abc^	36.35±2.58^abc^	0.15±0.023^abc^	6.92±0.45^abc^

Abbreviations: CME, coronary microembolization; LVEF, left ventricular ejection fraction; LVFS, left ventricular fractional shortening; CO, cardiac output; LVIDd, left ventricular internal diameter at end-diastole. Data are presented as mean ± SD. ^a^*P* < 0.05 versus the Sham group; ^b^*P* < 0.05 versus the CME group; ^c^*P* < 0.05 versus the CME+shRNA group.

### Inhibition of Egr-1 attenuated myocardial injury following CME

**Figure 2:**
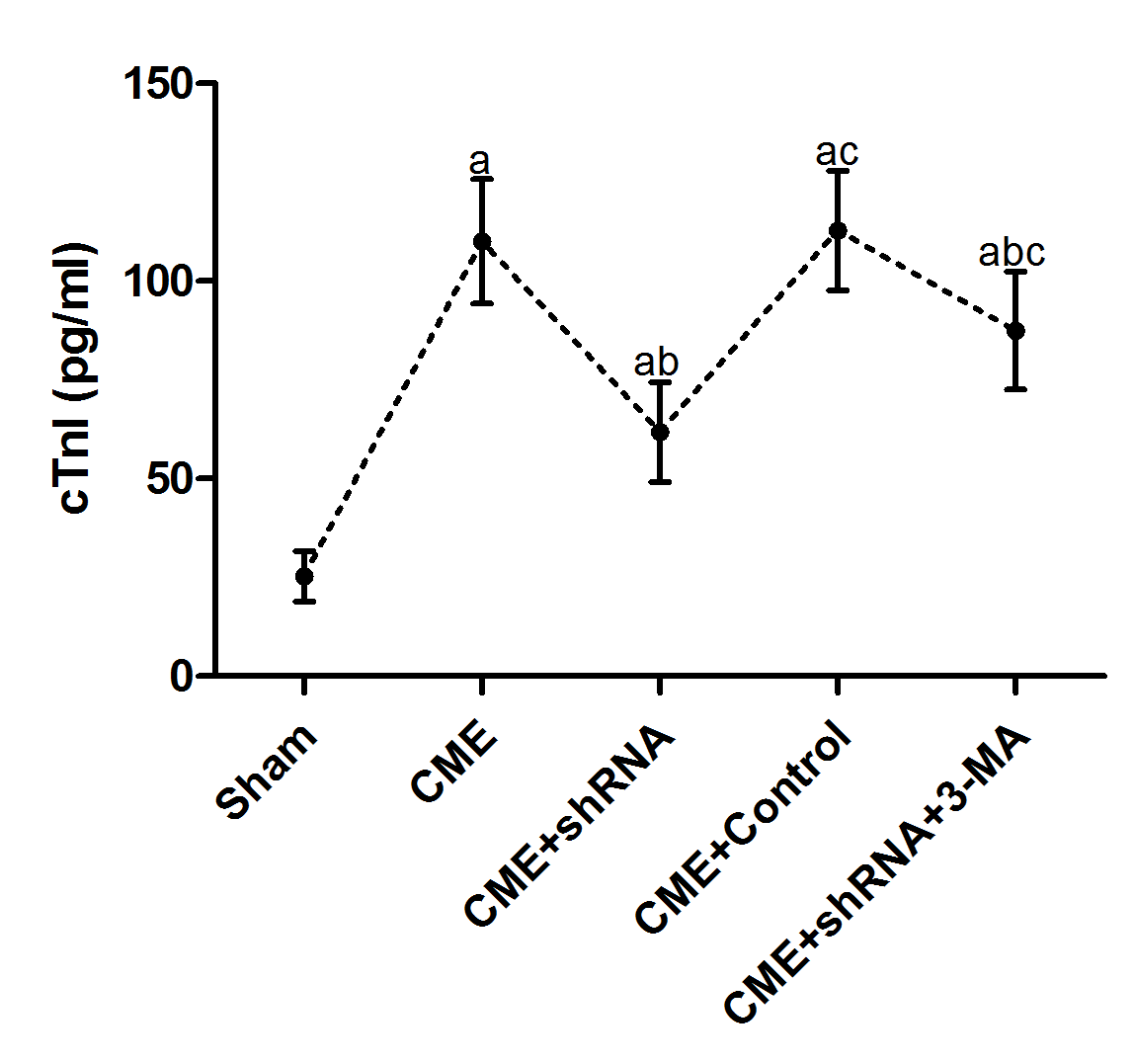
**Inhibition of Egr-1 attenuated myocardial injury following CME.** Egr-1 downregulation rapidly reduced the serum cTnI level after CME modeling. The results are presented as the means ± SD from at least three independent experiments. ^a^*P* < 0.05 compared with the Sham group; ^b^*P* < 0.05 compared with the CME group; ^c^*P* < 0.05 compared with the CME+shRNA group.

An ELISA was used to detect the changes in serum cTnI levels in each group. The results are presented in Table 2 and Fig. 2. The cTnI level in the CME group was significantly increased compared to the sham group (*P* < 0.05). Egr-1 downregulation rapidly reduced the serum cTnI level after CME modeling (*P* < 0.05). Pretreatment with 3-MA significantly increased the cTnI level compared with the Egr-1 downregulation group (*P* < 0.05).

**Table 2:** Serum cTnI concentrations of each group following CME modeling (pg/ml).

Group	*N*	cTnI
Sham	6	25.28±6.38
CME	6	110.08±15.76^a^
CME+shRNA	6	61.81±12.65^ab^
CME+Control	6	112.83±15.10^ac^
CME+shRNA+3-MA	6	87.45±14.93^abc^

Abbreviations: CME, coronary microembolization; cTnI, cardiac troponin I. Data are presented as mean ± SD. ^a^*P* < 0.05 versus the Sham group; ^b^*P* < 0.05 versus the CME group; ^c^*P* < 0.05 versus the CME+shRNA group.

### Inhibition of Egr-1 reduced myocardial microinfarct areas following CME

**Figure 3:**
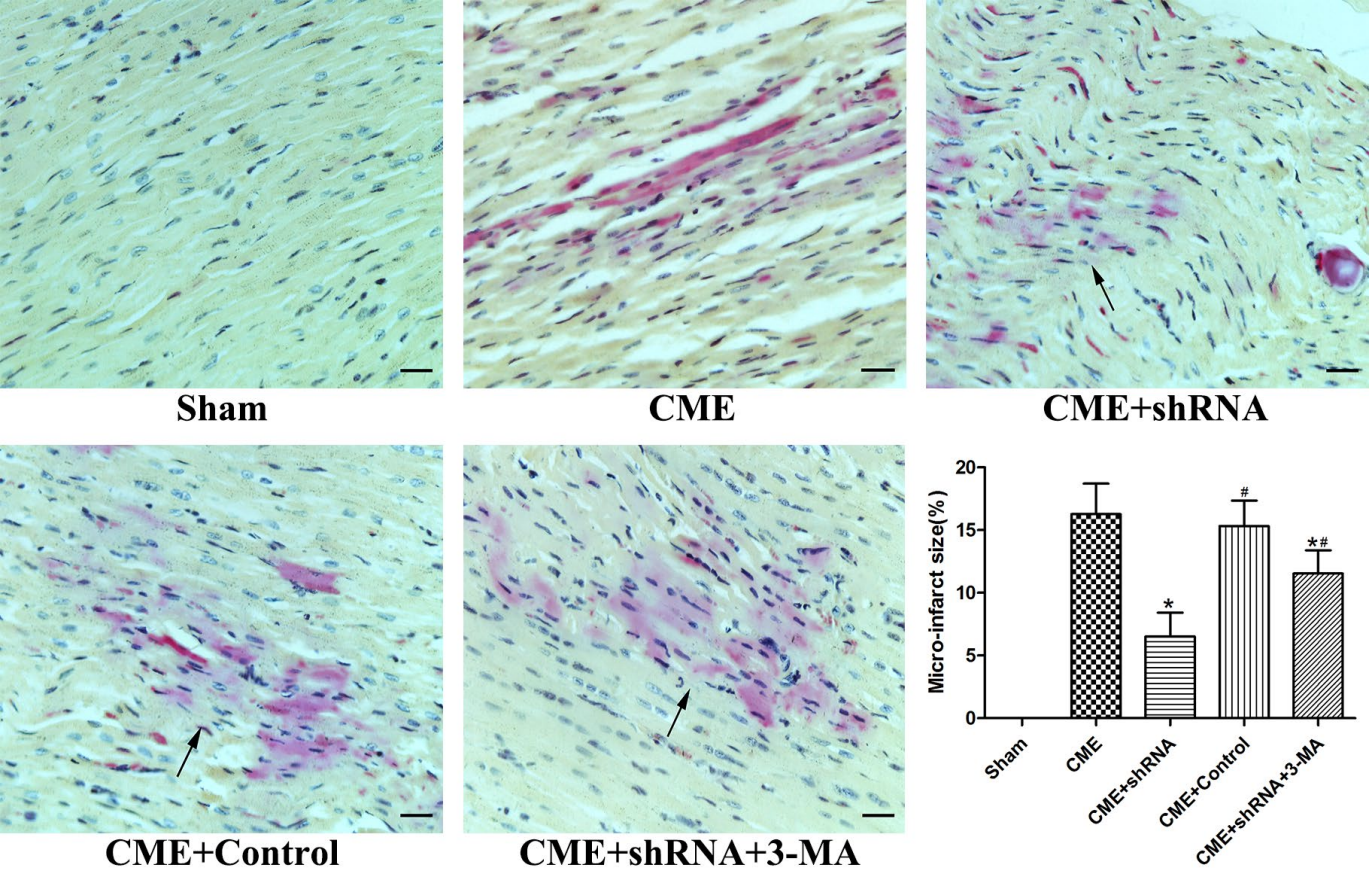
**Inhibition of Egr-1 reduced myocardial microinfarct areas following CME.** HBFP staining stained the normal myocardium yellow, while the ischemic myocardium was stained red. The arrow indicates the microinfarct focus (x400, scale bar = 25 μm). The results are presented as the means ± SD from at least three independent experiments. *P < 0.05 compared with the CME group; ^#^*P* < 0.05 compared with the CME+shRNA group.

HBFP staining was performed to measure the area of myocardial microinfarction (Fig. 3). No microinfarct areas were observed in the Sham group, while the microinfarct sizes of the CME, CME+Egr-1 shRNA, CME+Control shRNA, and CME+Egr-1 shRNA+3-MA groups were 16.28±2.43%, 6.52±1.91%, 15.33±2.02%, and 11.54±1.85%, respectively. Inhibition of Egr-1 significantly reduced the myocardial microinfarct area following CME (*P* < 0.05). Pretreatment with 3-MA significantly increased the microinfarction size compared to the Egr-1 downregulation group (*P* < 0.05).

### Inhibition of Egr-1 decreased myocardial AI following CME

**Figure 4:**
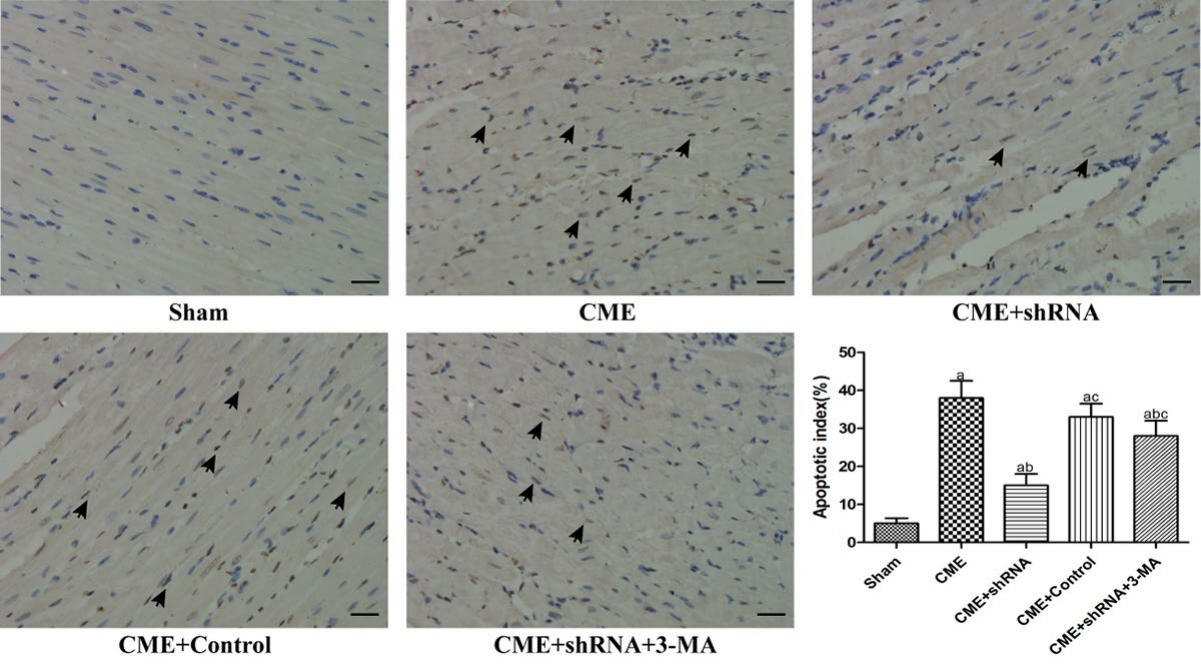
**Inhibition of Egr-1 decreased the myocardial AI following CME.** TUNEL staining of the microinfarcted myocardium stained the apoptotic nuclei yellow-brown, while the normal nuclei were stained light blue. The arrows indicate apoptotic nuclei (x400, scale bar = 25 μm). The results are presented as the means ± SD from at least three independent experiments. ^a^*P* < 0.05 compared with the Sham group; ^b^*P* < 0.05 compared with the CME group; ^c^*P* < 0.05 compared with the CME+shRNA group.

TUNEL staining was performed to detect myocardial AI. The myocardial AI in the sham, CME, CME+Egr-1 shRNA, CME+Control shRNA, and CME+Egr-1 shRNA+3-MA groups were 3.9±1.4%, 29.6±3.8%, 14.1±2.7%, 28.3±3.5%, and 23.5±3.1%, respectively (Fig. 4). The myocardial AI was significantly increased in the CME group compared to the sham group (*P* < 0.05). Inhibition of Egr-1 decreased the myocardial AI following CME (*P* < 0.05). Pretreatment with 3-MA significantly increased the AI compared with the Egr-1 downregulation group (*P* < 0.05).

### Inhibition of Egr-1 restored myocardial autophagy following CME

**Figure 5:**
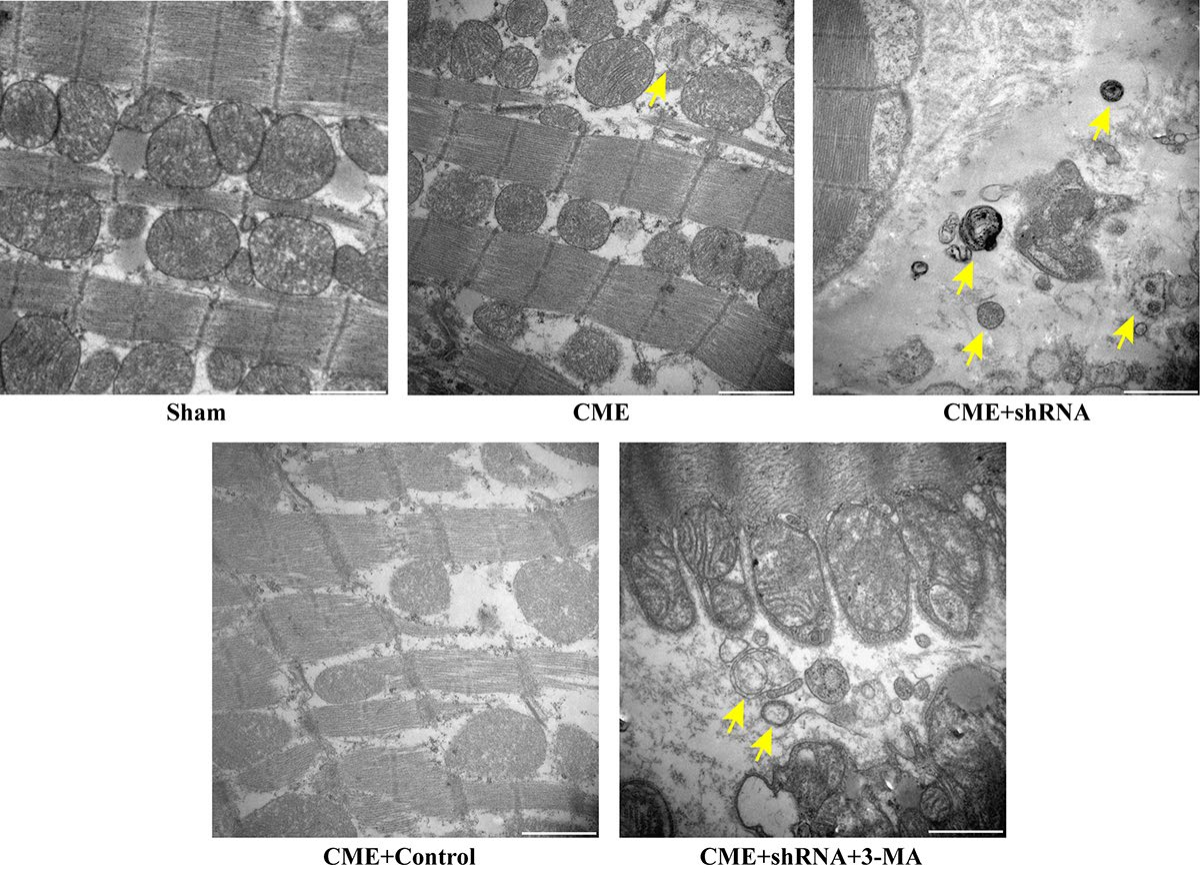
**Inhibition of Egr-1 restored myocardial autophagy following CME.** Image of autophagic vacuoles in the myocardium captured using a transmission electron microscopy. The arrow indicates typical autophagic vacuoles (x30,000, Scale bar = 1 μm).

The TEM observations showed normal ultrastructures of myofibrils and mitochondrial membranes in the Sham group. Myofibril fragmentation and mitochondrial swelling were visible in the CME group. The number of double-layer membrane structures characteristic of typical autophagic vacuoles was dramatically increased in the Egr-1 inhibitor group and significantly decreased in the 3-MA pretreatment group compared to the Egr-1 inhibitor group (Fig. 5). Thus, inhibition of Egr-1 restored myocardial autophagy following CME.

### Activation of the Egr-1/Bim/Beclin-1 pathway contributed to the CME-induced myocardial injury

**Figure 6:**
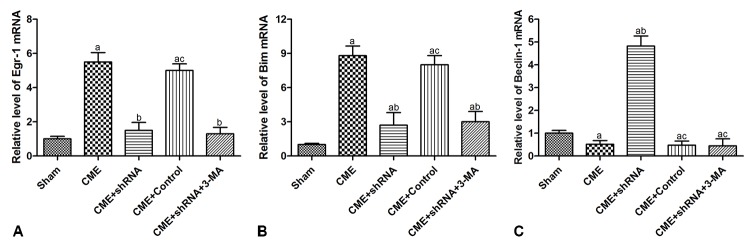
**Activation of the Egr-1/Bim/Beclin-1 pathway participated in CME-induced myocardial injury.** The expression of (**A**) the Egr-1, (**B**) Bim, and (**C**) Beclin-1 mRNAs in the myocardium of rat detected by RT-qPCR. The results are presented as the means ± SD from at least three independent experiments. ^a^*P* < 0.05 compared with the Sham group; ^b^*P* < 0.05 compared with the CME group; ^c^*P* < 0.05 compared with the CME+shRNA group.

RT-qPCR was conducted to detect the expression of mRNAs encoding intermediates in the Egr-1/Bim/Beclin-1 pathway in the rat myocardium (Fig. 6). The transfection of rAAV-Egr-1 shRNA via the tail vein successfully inhibited Egr-1 expression. Compared with the Sham group, the expression of the Egr-1 and Bim mRNAs were significantly increased in the CME group, while the Beclin-1 mRNA was decreased significantly (*P* < 0.05). Egr-1 downregulation reversed the effect of CME compared with the CME group (*P* < 0.05). Based on these results, activation of the Egr-1/Bim/Beclin-1 pathway contributed to CME-induced myocardial injury.

### The Egr-1/Bim/Beclin-1 pathway regulated autophagy and apoptosis during CME-induced myocardial injury

**Figure 7:**
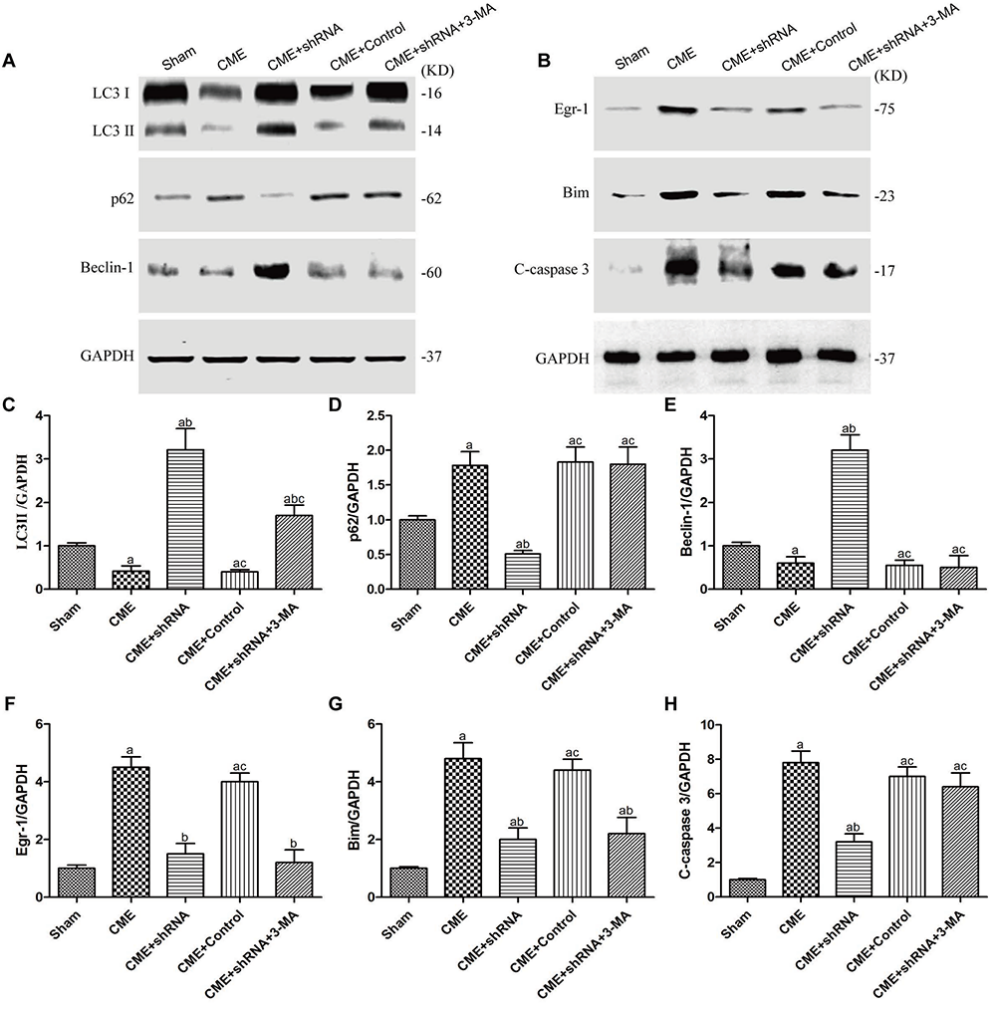
**The Egr-1/Bim/Beclin-1 pathway regulated autophagy and apoptosis during CME-induced myocardial injury.** (**A**) Representative western blots of autophagy-associated proteins. (**B**) Representative western blots of apoptosis-associated and Egr-1/Bim/Beclin-1 pathway proteins. c-h Levels of the (**C**) LC3-II, (**D**) p62, (**E**) Beclin-1, (**F**) Egr-1, (**G**) Bim, and (**H**) cleaved caspase-3 normalized to GAPDH. The results are presented as the means ± SD from at least three independent experiments. ^a^*P* < 0.05 compared with the Sham group; ^b^*P* < 0.05 compared with the CME group; ^c^*P* < 0.05 compared with the CME+shRNA group.

The levels of autophagy-associated proteins (LC3-II and Beclin-1) were reduced, and the level of an apoptosis-associated protein (cleaved caspase-3) was increased in the CME group (*P* < 0.05) (Fig. 7). Next, we confirmed the major role of the Egr-1/Bim/Beclin-1 signaling pathway in CME-induced myocardial injury. Western blot assays showed that Egr-1 silencing in rats substantially increased LC3-II and Beclin-1 levels, and decreased the cleaved caspase-3, p62, and Bim levels (*P* < 0.05). The blockade of the Egr-1/Bim/Beclin-1 pathway increased myocardial autophagy and decreased apoptosis. Pretreatment with 3-MA partially reversed these changes. Thus, the Egr-1/Bim/Beclin-1 pathway regulated autophagy and apoptosis during CME-induced myocardial injury.

## DISCUSSION

**Figure 8:**
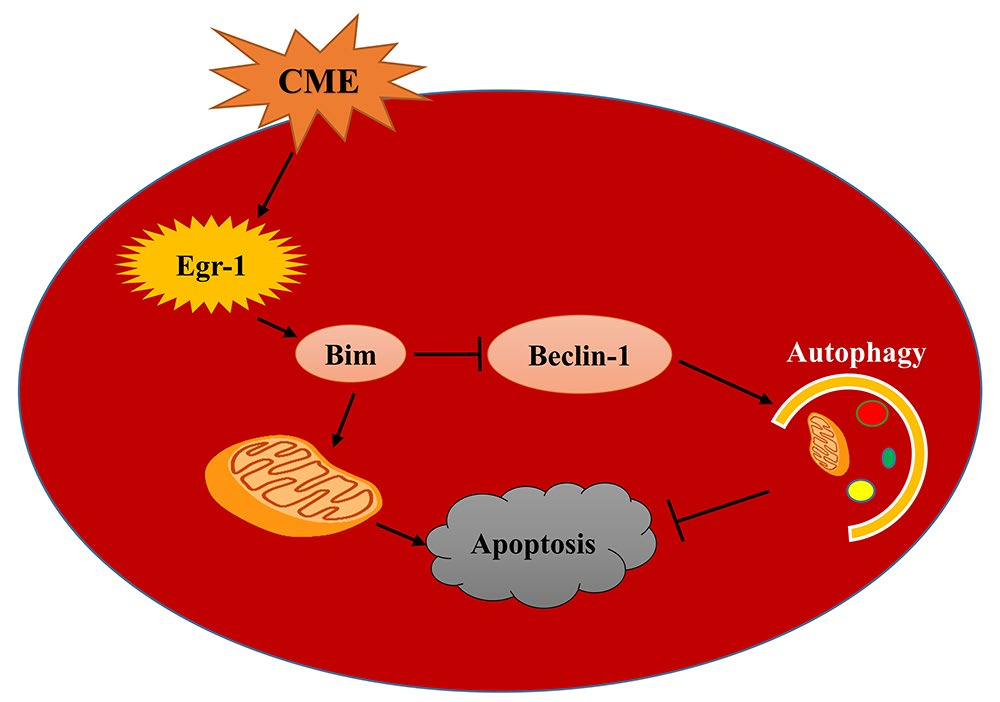
**A model of the mechanism underlying the functions of the Egr-1/Bim/Beclin-1 pathway in CME-induced myocardial injury.** CME triggers a complex cell death signaling pathway, including the upregulation of Egr-1, resulting in Bim activation and Beclin-1 inhibition, which inhibit myocardial autophagy and induce apoptosis to provoke myocardial injury and cardiac dysfunction. Based on the data reported in the present study, the Egr-1/Bim/Beclin-1 pathway may be involved in CME-induced myocardial injury by regulating myocardial autophagy and apoptosis.

In the present study, we investigated the activation of the Egr-1/Bim/Beclin-1 pathway after CME and its specific mechanism in mediating myocardial injury by constructing a CME model in rats. Myocardial injury, microinfarct areas and cardiac dysfunction are alleviated following Egr-1 inhibition in rats with CME. These benefits were reduced by a pretreatment with the autophagy inhibitor 3-MA. In the myocardium of the rat CME model, the Egr-1/Bim/Beclin-1 pathway was activated, accompanied by the inhibition of autophagy and increased apoptosis. The Egr-1/Bim/Beclin-1 pathway might be involved in CME-induced myocardial injury by regulating myocardial autophagy and apoptosis (Fig. 8).

CME is defined as coronary microcirculation thrombosis and microinfarction resulting from the spontaneous rupture of an atherosclerotic plaque or microemboli in individuals undergoing PCI [19]. CME may occur during reperfusion therapy, including thrombolytic therapy and PCI. Once CME occurs, it can result in a series of serious clinical complications, such as contractile dysfunction, malignant arrhythmias and even sudden cardiac death [3,20]. Many therapies have been employed in clinical practice, including mechanical strategies and pharmacological therapies, but the effects are not satisfactory [21].

The immediate-early gene product and zinc finger transcription factor Egr-1 plays a key master regulatory role in multiple cardiovascular pathological processes [14]. Egr-1 is expressed at high levels in human atherosclerosis, carotid plaques and neointimal lesions [15]. It is an attractive target for the therapeutic intervention of cardiovascular disease. Egr-1 directly transactivates Bim gene expression to induce apoptosis [16,17]. Other independent studies found that Bim exerts dual effects on inhibiting autophagy and promoting apoptosis, and Bim inhibits autophagy independent of its proapoptotic function [22–26]. Bim inhibits autophagy by directly interacting with Beclin-1, an important autophagy regulator. Thus, it may have important roles in disease pathogenesis. As shown in the present study, the Egr-1/Bim/Beclin-1 pathway was activated after CME; meanwhile, myocardial autophagy was dramatically inhibited and apoptosis was induced.

Myocardial autophagy is widely involved in the pathogenesis and development of cardiovascular diseases [27–29]. The inhibition of autophagy has been shown to exacerbate myocardial hypertrophy in patients with cardiac hypertrophy [30]. However, excess autophagy also results in autophagic cardiomyocyte death in patients with heart failure [31]. Impaired autophagy flux is associated with cardiomyocyte death following ischemia-reperfusion injury [32]. Strategies regulating autophagy flux are expected to be a novel therapeutic target for cardiovascular disease. In the present study, the reduction in the myocardial autophagy level was consistent with cardiac dysfunction after CME.

The present study investigated the possible mechanisms underlying CME-induced myocardial injury. CME-induced myocardial injury was associated with the activation of the Egr-1/Bim/Beclin-1 pathway, which inhibited myocardial autophagy and induced apoptosis. These findings may provide new therapeutic targets for myocardial injury in patients with CME-induced cardiac dysfunction.

The limitations of this study should be recognized. First, our findings were derived from an animal CME model, which was established by injecting plastic microspheres into the LV. Therefore, these results may not be directly comparable with those derived from in vivo microembolization. In clinical practice, the coronary microembolus is enriched with platelets, red blood cells and other bioactive factors. In the future, additional studies are needed to examine our findings in an animal model that more closely mimics human biology. Second, because autophagy is also regulated by many other complex pathways, we cannot exclude the possibility of interactions between Egr-1/Bim/Beclin-1 and other pathways. Further studies will be required to determine whether other autophagy-related pathways are also involved in CME-induced myocardial injury and the underlying mechanisms. Finally, the present study was only based on in vivo experiments, and we hope that the findings from the vivo experiments will be further confirmed at the cellular level in the near future.

In summary, the Egr-1/Bim/Beclin-1 pathway is activated in the myocardium after CME, accompanied by the inhibition of autophagy, induction of apoptosis, and cardiac dysfunction. Egr-1 is involved in CME-induced myocardial injury at least partially via Bim/Beclin-1 pathway-mediated suppression of autophagy and activation of apoptosis; thus, this pathway represents a potential therapeutic target in CME.

## MATERIALS AND METHODS

### Animal modeling and treatment

All in vivo experiments were performed in accordance with the guidelines of the Animal Care and Use Committee of Guangxi Medical University. Healthy adult male Sprague-Dawley (SD) rats (body weight 250-300 g) were purchased from Guangxi Medical Experimental Animal Center (Nanning, China). Thirty rats were randomly (random number) divided into five groups: sham, CME, CME+Egr-1 shRNA, CME+Control shRNA, and CME+Egr-1 shRNA+3-MA (n = 6 rats per group). We constructed a CME model in rats by injecting plastic microspheres into the left ventricle (LV), as previously described [33,34]. Briefly, a left thoracotomy was performed between the third and fifth intercostal spaces. The ascending aorta was completely exposed after dissecting the pericardium. During the 10 s occlusion of the ascending aorta, a suspension containing approximately 3,000 microspheres (42 μm in diameter, Biosphere Medical Inc., USA) in 100 μl of a saline solution was injected into the LV. Rats in the sham group were injected with the same dose of normal saline using the same procedure as the CME group. The CME+Egr-1 shRNA and CME+Control shRNA groups were transfected with Egr-1 shRNA and Control shRNA, respectively, by injections of a recombinant adeno-associated virus serotype 9 (rAAV9) through the tail vein for two weeks, and then the CME model was established. The CME+Egr-1 shRNA+3-MA group received an intraperitoneal injection of 3-MA (30 mg/kg) 30 minutes before establishing the CME model, and the other interventions were the same as the CME+Egr-1 shRNA group.

### Design, synthesis and transfection of the rAAV-Egr-1 shRNA

The rAAV-Egr-1 shRNA and rAAV-Control shRNA gene sequences were designed and synthesized by Hanbio Biotechnology (Shanghai, China). Two weeks before the CME modeling operation, 200 μl of a solution containing 4 x 10^11^ viral particles of rAAV-Egr-1 shRNA or rAAV-Control shRNA were injected through the tail vein as described previously [35,36]. Animals in the sham group were injected with saline. Two Egr-1 shRNAs were used to target the rat Egr-1 mRNA sequence: 5´-GGACTTAAAGGCTCTTAAT-3´ and 5´-GGACAAGAAAGCAGACAAA-3´. Control shRNA was as follows: 5´-TTCTCCGAACGTGTCACGTAA-3´.

### Heart function test

The heart function was tested by echocardiography using a Philips SONOS7500 instrument at a probe frequency of 10 MHz. The following parameters were evaluated: the left ventricular ejection fraction (LVEF), left ventricular fractional shortening (LVFS), cardiac output (CO) and left ventricular internal diameter at end-diastole (LVIDd). All echocardiographic evaluations in each group were performed by a professional physician who was blinded to the treatments, and the results are presented as the average of three heart beat cycles.

### Measurement of serum cardiac troponin I (cTnI) levels

EDTA anticoagulant-treated blood samples were collected from the abdominal aorta before each rat was sacrificed. Serum samples were added to a 96-well plate, and then the concentration of cTnI in serum was determined using a rat cTnI Enzyme-linked Immuno Sorbent Assay (ELISA) kit (Roche, Inc., Switzerland) according to the manufacturer’s instructions. The optical density (OD) value was read at 450 nm using a light absorption microplate reader. After establishing a standard curve based on the absorbance readings, the levels of cTnI in samples were calculated from the OD values.

### Tissue preparation and pathological examination

After heart function measurements were performed, the rats were immediately euthanized. The myocardial tissue was collected after an infusion with cold normal saline. Half of the ventricles were then isolated from the hearts and stored at -80°C after freezing in liquid nitrogen for molecular biology experiments. The other half of the ventricles were fixed with 4% paraformaldehyde at 4°C for 12 h, and then embedded in paraffin. Afterwards, the paraffin-embedded myocardial tissue was serially sectioned at a thickness of 4 μm for hematoxylin-basic fuchsin-picric acid (HBFP) staining and terminal-deoxynucleotidyl transferase-mediated nick end labeling (TUNEL) staining.

### Measurement of the myocardial microinfarct area

Early myocardial ischemia and infarct regions are recognized by HBFP staining. The normal myocardium was stained yellow or brown, while the ischemic or infarcted myocardium was stained red. The histological analysis of HBFP-stained sections was conducted with a DMR-Q550 pathological image analyzer (Leica, Germany). Briefly, five x200 magnification visual fields were randomly selected from each section in four sections per sample for observation using the Leica Qwin image analysis software. The microinfarct area was measured using planimetry, and the results are presented as percentages of the total analyzed areas.

### Measurement of the myocardial apoptotic index (AI)

A TUNEL assay kit (Roche, USA) was used to detect cardiomyocyte apoptosis. TUNEL staining was performed strictly according to the manufacturer’s instructions. The normal nuclei were light blue, while the apoptotic nuclei were yellow-brown. Ten x400 magnification visual fields were randomly selected from each slice to count TUNEL-positive nuclei. The myocardial AI was calculated using the following formula: the number of TUNEL positive nuclei / total nuclei x100%.

### Transmission Electron Microscopy (TEM)

The ultrastructure of the myocardial tissue, including the myofibrillar structure, mitochondrial membrane and typical autophagic vacuoles, was observed using a TEM. Myocardial tissue samples from each group were cut into 1 mm x 1 mm x 1 mm pieces and then fixed with 2.5% glutaraldehyde at 4°C overnight. Afterwards, the specimens were sequentially treated as follows: cleaned, dehydrated, embedded, sliced, stained, and observed. All examinations were conducted using a TEM (Hitachi H-7650, Japan) under at x30,000 magnification by an experienced pathologist who was blinded to the groups.

### Quantitative real-time PCR

Total RNA was extracted with the TRIzol reagent (Gibco, USA) according to the protocols supplied by the manufacturer. The concentration of RNA was measured using a NanoDrop spectrophotometer (Thermo Fisher Scientific Inc., USA) and then subjected to reverse transcription using PrimeScript RT reagent kit (TaKaRa, Japan). RT-qPCR was performed using the ABI PRISM 7500 system (Applied BioSystems, USA) with SYBR Premix Ex Taq kit (TaKaRa, Japan). PCR primer sequences were designed and synthesized by TaKaRa Biotechnology (Dalian, China), as listed in Table 3. The relative changes in mRNA levels were calculated using the 2^-∆∆Ct^ method and were normalized to β-actin.

**Table 3:** Primer sequences.

Gene	Primer sequences (5´-3´)
Egr-1 forward	GAACAACCCTACGAGCACCTG
Egr-1 reverse	GCCACAAAGTGTTGCCACTG
Bim forward	TAAGTTCTGAGTGTGACCGAGA
Bim reverse	GCTCTGTCTGTAGGGAGGTAGG
Beclin-1 forward	TTGGCACAATCAATAACTTCAGGC
Beclin-1 reverse	CCGTAAGGAACAAGTCGGTATCTC
β-actin forward	ATTGCCGACAGGATGCAGAA
β-actin reverse	CAAGATCATTGCTCCTCCTGAGCGCA

### Western blots

Protein was extracted from the myocardium using a Protein Extraction Kit (Solarbio, China). The concentration of protein was measured using the bicinchoninic acid (BCA) method (Beyotime Biotechnology, China). The extracted protein (50 μg) was separated on 10% or 15% SDS polyacrylamide gels and then transferred to PVDF membranes (Millipore). After blocking for 1 h at room temperature with 5% fat-free milk in TBS, membranes were incubated overnight at 4°C with the following primary antibodies: LC3-II, p62, Egr-1, Bim, Beclin-1, and GAPDH (all from CST, USA). Membranes were incubated with the appropriate secondary antibodies (Abcam, USA) in dark for 2h at room temperature after 3 washes with TBST. Finally, an Odyssey infrared fluorescence scanning imaging system (Odyssey, LICOR, USA) was used to analyze the intensity of the protein bands. The relative changes in protein levels were normalized to GAPDH.

### Statistical analysis

All data are presented as the mean values ± standard deviations. Differences between multiple groups were analyzed using one-way analysis of variance (ANOVA) with SPSS 21.0 (SPSS Inc., USA). All experiments were performed independently and in triplicate. Values of *P* < 0.05 were considered to indicate a statistically significant difference.
